# Book Notices

**Published:** 1897-08

**Authors:** 


### Book Notices.

---

THE Medical Gazette Publishing Co., of Cleveland, Ohio, announces a small volume to be issued soon with the title "About Children." The author is Dr. Samuel W. Kelley, of the Cleveland College of Physicians and Surgeons. The book will contain six lectures filled with information for nurses, medical practitioners, students and all who have the care of children. Advance orders will be filled in September.

---

THE editor of the *American Monthly Review of Reviews*, in his department entitled "The Progress of the World," discusses harvest and trade prospects, the new tariff, the coal strike, American annexation policies, our diplomacy on the seal question, Japan and Hawaii, British interests in Canada, European politics, and many other timely topics. In connection with matter on the Klondyke gold fields an excellent map of Alaska is published. In the same department appear interesting views of important British colonial capitals.

---

DR. BYRON ROBINSON'S book on the Peritoneum, its Histology and Physiology, Vol. I., will be ready for the market in August. It will contain 240 illustrations, 150 of which are of his own drawing. The book contains 400 pages of the exact size of Quain's New Anatomy. The bibliography of the peritoneum added to the book is the largest in the world, being larger than the book itself. The book is a product of his own original researches. Practitioners and abdominal surgeons will find in it a full knowledge of the physiology and histology of the peritoneum up to date. Dr. Robinson is now engaged in completing a small book, which will soon be issued, on the Abdominal Brain and Automatic Visceral Ganglia.

---

Surgical Hints for the Surgeon and General Practitioner. By Howard Lilienthal, M. D., Assistant Attending Surgeon to Mt. Sinai Hospital, New York City, New York: International Journal of Surgery Company, 1897. Price, 25 cents.

In writing this little book the author's aim has been to present a number of observations and suggestions whose value has been thoroughly tested at the bedside and in the operating room. A review of its pages will show how much practical information he has conveyed within a small compass, and this he has been able to do by eschewing all superfluous verbiage and by writing clearly and to the point. The material is well arranged, the typography excellent, and the little volume is of a convenient size to be carried in the pocket and perused at leisure moments.

---

General Harrison's Book.—*The Story Why Mr. Bok Released all Claims to Royalty.*—The Indianapolis *Journal* prints this interesting story concerning ex-President Harrison's forthcoming book: General Harrison has just completed the revision of his articles which have appeared in *The Ladies' Home Journal*, making extended notes and additions to them. There is a little story in connection with both articles and publication. When the arrangement for the articles was made with General Harrison by Edward W. Bok, editor of *The Ladies' Home Journal*, the General was paid for them, with the understanding that when they were put into book form the magazine was to share the royalties accruing therefrom. Mr. Bok, however, of his own accord, generously released General Harrison from paying him any royalty, for the reason, as he states, that by the publication of the articles by General Harrison the subscription list of his magazine was enlarged many thousands. The profits to

*The Ladies' Home Journal* were more than the publishers anticipated, and in view of this Mr. Bok asks nothing further. General Harrison placed the disposition of his book in Mr. Bok's hands. The best offer came to the editor from the Scribners, and to them Mr. Bok gave the book for his distinguished contributor. General Harrison's revision of the book has just been completed, and the volume will appear in the autumn.

